# Comparative pharmacovigilance of non-benzodiazepine receptor agonists versus dual orexin receptor antagonists for insomnia in older adults

**DOI:** 10.3389/fphar.2026.1788736

**Published:** 2026-03-10

**Authors:** Shuqing Gao, Xiaotang Feng, Qi Li, Jingyi Fan

**Affiliations:** Department of Psychiatry, Nanjing Youan Hospital, Nanjing, China

**Keywords:** DORAs, FAERS, insomnia, nBZRAs, older adults

## Abstract

**Background:**

The accelerating global aging trend has positioned insomnia in the elderly as a major public health challenge. Although cognitive behavioral therapy is the first-line intervention, pharmacological treatment remains widely used. However, significant safety concerns exist regarding the use of traditional non-benzodiazepine sedative-hypnotics (nBZRAs) in older adults, while long-term real-world safety evidence for newer dual orexin receptor antagonists (DORAs) remains scarce. Addressing this evidence gap is critical for guiding safe medication use in the aging population.

**Methods:**

We conducted a pharmacovigilance study utilizing the U.S. Food and Drug Administration Adverse Event Reporting System (FAERS) database, covering reports from the first quarter of 2004 to the second quarter of 2025. We included reports for patients aged ≥65 years where a target nBZRA or DORA was listed as the primary suspect drug. A comprehensive disproportionality analysis was performed using the Reporting Odds Ratio (ROR), Proportional Reporting Ratio (PRR), Information Component (IC), and Empirical Bayesian Geometric Mean (EBGM), with stringent thresholds to minimize false positives.

**Results:**

A total of 5,447 reports for elderly patients were analyzed. The study revealed distinct adverse event profiles between the two drug classes. nBZRAs, particularly eszopiclone, showed the strongest signals related to therapeutic failure (e.g., “Drug ineffective,” “Insomnia”) alongside a unique signal for “Dysgeusia”. In contrast, DORAs exhibited strong and consistent signals for “Dream-abnormality” events concordant with their sleep-wake modulation mechanism, including “Nightmare,” “Abnormal dreams,” and “Hallucination”. Notably, none of the studied drugs generated a statistically significant signal for “Fall” within this dataset. System Organ Class analysis showed that psychiatric and nervous system disorders had the highest incidence.

**Conclusion:**

These findings highlight distinct safety profiles: nBZRAs are linked to therapeutic failure and dysgeusia, while DORAs are associated with neuropsychiatric events such as nightmares and hallucinations.

## 1 Introduction

The world is currently undergoing a period of rapid population aging. In recognition of this demographic shift, the United Nations General Assembly has declared 2021–2030 the “Decade of Healthy Aging” (Chen et al., 2022), underscoring the urgent need to address age-related health challenges. Among these, insomnia represents a highly prevalent public health issue in the elderly population, generally defined as individuals aged 65 years and older. Its incidence shows a marked increase with advancing age (Abad and Guilleminault, 2018; Brewster et al., 2022; Gulia and Kumar, 2018). The elevated risk of insomnia in older adults stems from a confluence of factors spanning environmental, behavioral, psychological, physiological, and social domains (Brewster et al., 2022; Morin and Buysse, 2024). Besides, insomnia in older adults is rarely an isolated condition; it frequently co-occurs with psychiatric disorders and polypharmacy, which complicates both treatment and safety attribution in observational data (Fornaro et al., 2024a). While insomnia may initially be situational or transient, more than 50% of cases evolve into a persistent, chronic condition (Morin and Buysse, 2024). Chronic insomnia extends beyond impaired nighttime rest; it is significantly associated with diminished quality of life, cognitive decline, mood disorders, and an increased risk of cardiovascular events (Bertisch et al., 2018; Hertenstein et al., 2019; Shi et al., 2018). Reviews indicate that over half of older adults in the United States are affected by chronic insomnia, with women being approximately 50% more susceptible than men. This trend of high prevalence and greater risk among women is corroborated by studies in other countries, confirming its global significance (Abad and Guilleminault, 2018).

Therefore, active intervention for insomnia in the elderly population is particularly necessary. Treatment options include both non-pharmacological and pharmacological approaches. Given its favorable safety and efficacy profile, durable effects, and cost-effectiveness (Beaulieu-Bonneau et al., 2017; van Straten et al., 2018). Cognitive Behavioral Therapy for Insomnia (CBT-I) is recommended as first-line treatment (Abad and Guilleminault, 2018; Brewster et al., 2022; Sutton, 2021). Pharmacotherapy may be considered when CBT-I is insufficient or not feasible (Riemann et al., 2023). Pharmacological options can be categorized into benzodiazepines receptor agonists (BZRAs), non-benzodiazepine receptor agonists (nBZRAs, also known as Z-drugs), dual orexin receptor antagonists (DORAs), and other agents (such as low-dose antidepressants, antipsychotics, antihistamines, etc.) (Riemann et al., 2023). Although BZRAs and nBZRAs are frequently prescribed for insomnia management (Cooke and Ancoli-Israel, 2011; Enomoto et al., 2010; Lukačišinová et al., 2021; Matheson and Hainer, 2017; Olfson et al., 2015; Schroeck et al., 2016), their use in older patients is limited due to significant safety concerns, particularly for BZRAs. These risks include adverse effects such as sedation, impaired motor coordination, falls, fractures, cognitive impairment, and hospitalization (“American Geriatrics Society 2015 Updated Beers Criteria for Potentially Inappropriate Medication Use in Older Adults,” American Geriatrics Society 2015 Beers Criteria Update Expert Panel, 2015; Cooke and Ancoli-Israel, 2011; Glass et al., 2005; Pagel and Parnes, 2001; Schroeck et al., 2016; Tannenbaum, 2015). Long-term use may further lead to tolerance, dependence, withdrawal symptoms, and rebound insomnia (Brewster et al., 2022; Kamel and Gammack, 2006; Patel et al., 2018; Schroeck et al., 2016). Consequently, these drugs are included in the Beers Criteria for potentially inappropriate medication use in older adults (“American Geriatrics Society 2023 updated AGS Beers Criteria® for potentially inappropriate medication use in older adults,” American Geriatrics Society Beers Criteria® Update Expert Panel, 2023). However, a significant gap persists between clinical practice and guideline recommendations. For instance, utilization rates of these medications remain high among the elderly population, with benzodiazepine use ranging from 5.3% to 10.8% in individuals aged 50 years and older (Maust et al., 2019). This underscores the need for safer treatment alternatives.

To address the limitations of traditional sedative-hypnotics, a new class of DORAs have emerged. The orexin signaling system (comprising orexin-A and orexin-B, along with their G protein-coupled receptors OX1R and OX2R) plays a central role in the physiological regulation of sleep-wake cycles (Yin et al., 2015). Orexin receptor antagonists selectively bind to OX1R and/or OX2R, exerting competitive antagonism that promotes sleep and improves insomnia. Their safety and efficacy have been validated, making DORAs a promising treatment option for insomnia, potentially filling a therapeutic gap for patients with inadequate response to cognitive behavioral therapy or other insomnia medications (Wu et al., 2022). Among them, DORAs such as lemborexant have shown positive prospects in randomized controlled trials (RCTs) involving elderly patients with insomnia, demonstrating effectiveness in improving both sleep onset and maintenance with favorable short-term tolerability (Arnold et al., 2024; Rosenberg et al., 2019). These rigorous RCT findings provide preliminary support for the use of DORAs in the elderly population.

However, it should be noted that while RCTs represent the gold standard for establishing efficacy, caution is warranted when extrapolating their conclusions to real-world clinical practice in the elderly. Due to ethical and methodological requirements, RCTs typically employ strict inclusion and exclusion criteria, resulting in a highly homogeneous study population. They often exclude “typical” older patients who present with multiple comorbidities, complex concomitant medications, moderate-to-severe cognitive impairment, or other sleep disorders—precisely the patients most commonly encountered in clinical practice. Furthermore, RCTs are generally characterized by limited sample sizes and relatively short observation periods. This design makes it difficult to detect rare or delayed adverse events, as well as subtle risks that are particularly relevant to the elderly population. Most notably, there is currently a relative lack of direct, head-to-head evidence comparing the safety profiles of DORAs with the widely used yet potentially risky nBZRAs within this specific population.

Therefore, to address this critical evidence gap, there is an urgent need to utilize large-scale real-world data (RWD) to comprehensively evaluate and compare the safety profiles of nBZRAs and DORAs in the elderly population. The U.S. Food and Drug Administration Adverse Event Reporting System (FAERS), with its “large-scale” and “spontaneous reporting” characteristics, is well-suited for detecting rare and delayed adverse reactions that are difficult to identify in RCTs. Furthermore, its data are derived from a highly heterogeneous real-world patient population. This study aims to systematically mine and compare the characteristics and signal strengths of adverse event reports associated with the use of nBZRAs and DORAs in elderly patients, with the goal of identifying, quantifying, and contrasting their potential risks. The findings are expected to provide critical real-world evidence to support individualized and safer medication decision-making for insomnia in the elderly.

## 2 Methods

### 2.1 Data source and extraction

The data for this study were obtained from the publicly available ASCII-format data files of the FAERS database, covering the period from the first quarter of 2004 to the second quarter of 2025 (a total of 86 quarters). The data files were downloaded from the official website (https://fis.fda.gov/extensions/FPD-QDE-FAERS/FPD-QDE-FAERS.html) and include multidimensional information such as patient demographics, drug details, adverse event reports, therapeutic indications, and outcomes. All quarterly data were uniformly stored and managed using the MySQL database management system to ensure data integrity and traceability.

### 2.2 Data standardization and cleaning

Due to the evolution of the FAERS database over many years, significant variations exist in the storage formats, coding standards, and data structures across different quarters. A systematic data standardization process was performed: using the data format from the second quarter of 2025 as the reference, all historical data were standardized through detailed field mapping and conversion rules. This included unifying date formats, normalizing drug name spellings, and standardizing adverse event codes. This process was implemented via Python-automated scheduling of SQL scripts, ensuring consistency and comparability of the data spanning 20 years. Data cleaning followed a multi-step workflow: first, the demographic table (DEMO) was linked with the drug information table (DRUG) using INNER JOIN, followed by LEFT JOIN operations to integrate the adverse event table (REAC), indication table (INDI), and outcome table (OUTC). Only reports from patients aged 65 years or older were retained. To address duplicate reports, a strict deduplication strategy was applied: for reports sharing the same CASEID, the record with the largest PRIMARYID was kept; records with duplicate PRIMARYIDs as well as those listed in the FDA’s official deletion list were fully excluded. Additionally, records containing spelling errors, unrecognizable, or invalid information were removed.

### 2.3 Study drugs and adverse event definitions

**FIGURE 1:**
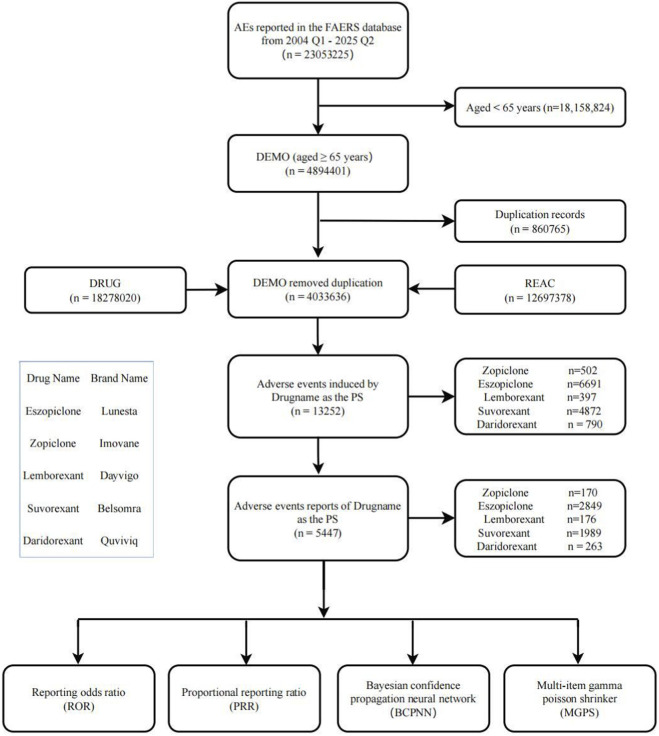
Data filtering flowchart.

This study focused on comparing the safety profiles of two classes of insomnia medications in the elderly population (≥65 years): nBZRAs (eszopiclone/Lunesta and zopiclone/Imovane) and DORAs (lemborexant/Dayvigo, suvorexant/Belsomra, and daridorexant/Quviviq). Target drug reports were accurately identified by searching for both generic and brand names of these medications, restricting the drug role code (ROLE_COD) to “PS” (Primary Suspect Drug). All adverse events were coded and classified using the Medical Dictionary for Regulatory Activities (MedDRA) version 26.0 (Tieu and Breder, 2018) and were analyzed at both the Preferred Term (PT) and System Organ Class (SOC) levels. An overview of the streamlined study process is presented in Figure 1.

### 2.4 Statistical analysis

A comprehensive disproportionality analysis was employed to evaluate the strength of association between the drugs and adverse events (AEs), utilizing multiple algorithms: the Reporting Odds Ratio (ROR) (Noguchi et al., 2020), the Proportional Reporting Ratio (PRR) (Evans et al., 2001), the Information Component (IC) from the Bayesian Confidence Propagation Neural Network (BCPNN) (Robert et al., 2023), and the Empirical Bayesian Geometric Mean (EBGM) (Jiang et al., 2024; Noguchi et al., 2021). These methods combine the advantages of frequentist and Bayesian statistics. These methods offer better control over reporting biases and the influence of comorbidities, meanwhile reduce sensitivity in the analysis of rare events.

A conservative threshold for positive signal detection was established to minimize false-positive results: the lower limit of the 95% confidence interval (95% CI) for ROR >1 (with a minimum of 3 cases), PRR ≥ 2 with a χ^2^ ≥ 4 (with a minimum of 3 cases), the lower limit of the 95% CI for IC > 0, and the lower limit of the 95% CI for EBGM > 2 (Jiang et al., 2024; Liu et al., 2024).

The technical implementation adopted a multi-layered architecture. The MySQL database served as the core for data storage, handling the storage and querying of large-scale data. Python was responsible for workflow scheduling and automation management, coordinating the various processing stages. SQL statements executed the specific data processing logic. The final data analysis, statistical computation, and visualization were performed using a combination of tools including Python, and Excel, ensuring the accuracy of the analytical results and the quality of visual presentations. All data processing and analyses adhered to the best practice standards for pharmacovigilance research.

## 3 Results

### 3.1 Basic information of study population

**FIGURE 2:**
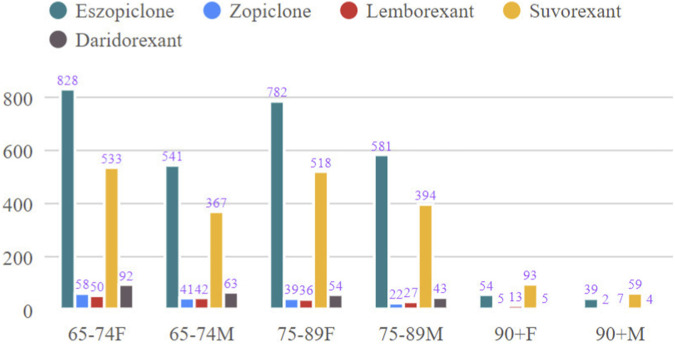
Age and sex distribution of adverse event reports across drug groups. Data are sourced from the FAERS database (2004Q1–2025Q2), including only patients aged ≥65 years. The grouped bar chart displays the number of reports for males and females within different age strata (65–74, 75–89, ≥90 years) for each drug group. F: female, M: male, 90+: ≥90 years.

**FIGURE 3:**
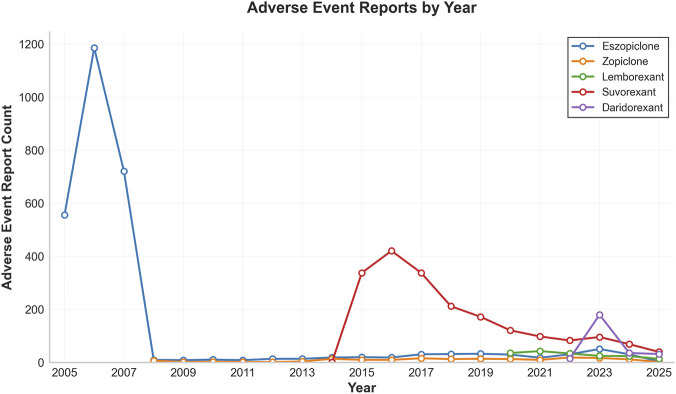
Temporal trends in the quarterly number of adverse event reports for each drug. The x-axis represents the year and quarter of report receipt, and the y-axis represents the number of reports. The line chart illustrates the quarterly count of adverse event reports for the five studied drugs over the observation period.

A total of 5,447 adverse event reports for elderly patients (≥65 years) were included. The age distribution was balanced, with the majority concentrated between 65 and 89 years. Female patients slightly outnumbered males across all drug groups. Differences were observed in reporter type and geographic distribution: eszopiclone and daridorexant reports were predominantly from consumers, while lemborexant and suvorexant had higher proportions of physician reports; most reports originated from the United States and Japan. Regarding fatal outcomes, the zopiclone group had the highest proportion (13.5%). The time-to-onset profiles differed: most events for eszopiclone and daridorexant occurred within 30 days of use, whereas over half of the events for suvorexant were reported after 30 days of medication use (Table 1). Further characterization of adverse event reporting patterns across drug groups is presented through visual analyses. As shown in Figure 2, variations exist in the age and sex distributions among different drug cohorts. Figure 3 illustrates the temporal trends in the volume of reported adverse events, indicating the reporting activity levels for each drug during various post-marketing periods. Corresponding detailed numerical data are provided in Supplementary Tables S1 and S2.

**TABLE 1:** Clinical characteristics of the study population (n = 5,447).

Characteristic	Eszopiclone	Zopiclone	Lemborexant	Suvorexant	Daridorexant
	n = 2,849	n = 170	n = 176	n = 1,989	n = 263
Age					
65–74	1,384 (48.6%)	102 (60%)	92 (52.3%)	912 (45.9%)	155 (58.9%)
75–89	1,371 (48.1%)	61 (35.9%)	64 (36.4%)	922 (46.4%)	98 (37.3%)
≥90	94 (3.3%)	7 (4.1%)	20 (11.4%)	155 (7.8%)	10 (3.8%)
Sex					
Male	1,161 (40.8%)	65 (38.2%)	76 (43.2%)	820 (41.2%)	110 (41.8%)
Female	1,664 (58.4%)	102 (60%)	99 (56.3%)	1,144 (57.5%)	151 (57.4%)
Missing	24 (0.8%)	3 (1.8%)	1 (0.6%)	25 (1.3%)	2 (0.8%)
Reporter type					
Consumer	2,372 (83.3%)	101 (59.4%)	53 (30.1%)	1,062 (53.4%)	197 (74.9%)
Physician	174 (6.1%)	33 (19.4%)	64 (36.4%)	655 (32.9%)	40 (15.2%)
Other	173 (6.1%)	31 (18.2%)	52 (29.5%)	256 (12.9%)	26 (9.9%)
Missing	130 (4.6%)	5 (3%)	7 (4.0%)	16 (0.8%)	0
Reporter country					
America	2,698 (94.7%)	125 (73.5%)	83 (47.2%)	1,539 (77.4%)	220 (83.7%)
Japan	135 (4.7%)	12 (7.1%)	76 (43.2%)	445 (22.4%)	8 (3.0%)
Other	11 (0.4%)	32 (18.8%)	17 (9.7%)	5 (0.3%)	35 (13.3%)
Missing	5 (0.2%)	1 (0.6%)	0	0	0
Death outcome	34 (1.2%)	23 (13.5%)	11 (6.3%)	58 (2.9%)	12 (4.6%)
Onset time					
0–30 d	2,122 (74.5%)	42 (24.7%)	75 (42.6%)	775 (39.0%)	132 (50.2%)
≥30 d	195 (6.8%)	12 (7.1%)	8 (4.5%)	1,162 (58.4%)	10 (3.8%)
Missing	532 (18.7%)	116 (68.2%)	93 (52.8%)	52 (2.6%)	121 (46.0%)

Data are presented as n (%). Percentages may not sum to 100% due to rounding or missing values. d, days.

### 3.2 Adverse reaction signal profiles of nBZRAs

Among the non-benzodiazepine drugs, eszopiclone (n = 2,849) generated the highest number of adverse event reports. As shown in Table 2, its strongest signals were concentrated on PTs such as “Drug Ineffective,” “Insomnia,” “Dysgeusia,” and specific insomnia subtypes (“Middle Insomnia” and “Initial Insomnia”). All four disproportionality analysis metrics for these PTs indicated exceptionally strong signal strengths (e.g., ROR for “Initial Insomnia” was 399.43, 95% CI: 352.90–452.10; for “Middle Insomnia” was 282.48, 95% CI: 255.72–312.04), strongly suggesting that the association of these events with eszopiclone use far exceeds background expectation. It is noteworthy that signals for typical sedative-hypnotic related adverse reactions such as “Somnolence,” “Dizziness,” and “Headache” were relatively moderate, while “Nausea” showed no significant signal (ROR: 0.98, 95% CI: 0.78–1.22; IC025 < 0). Zopiclone had a smaller total number of reports (n = 170), but its signal profile revealed different safety concerns. In addition to “Drug Ineffective” and “Insomnia,” signals emerged for “Product Quality Issue,” “Product Substitution Issue,” and the serious outcome of “Completed Suicide.” The signal for “Completed Suicide” was strong (ROR: 35.45, 95% CI: 21.54–58.35).

**TABLE 2:** Top 10 PTs with signal strengths for adverse events associated with nBZRAs.

Drug name	PT	n	ROR (95% Cl)	PRR (χ^2^)	EBGM (EBGM05)	IC (IC025)
Eszopiclone	Drug ineffective	1,415	19.36 (18.26–20.54)	15.48 (19,276.45)	15.36 (14.48)	3.94 (3.84)
	Insomnia	1,009	48.73 (45.54–52.13)	41.53 (39,199.56)	40.66 (38.00)	5.35 (5.19)
	Dysgeusia	598	74.86 (68.73–81.53)	68.26 (38,305.32)	65.92 (60.53)	6.04 (5.77)
	Middle insomnia	471	282.48 (255.72–312.04)	262.67 (107,874.78)	230.84 (208.97)	7.85 (7.13)
	Initial insomnia	313	399.43 (352.90–452.10)	380.80 (98,754.52)	317.29 (280.33)	8.31 (7.12)
	Somnolence	172	8.36 (7.18–9.73)	8.17 (1,080.69)	8.14 (6.99)	3.02 (2.74)
	Dizziness	119	1.93 (1.61–2.31)	1.91 (52.20)	1.91 (1.59)	0.93 (0.66)
	Headache	113	2.33 (1.93–2.80)	2.30 (83.87)	2.30 (1.91)	1.20 (0.91)
	Nausea	77	0.98 (0.78–1.22)	0.98 (0.04)	0.98 (0.78)	−0.03 (−0.36)
	Drug effect decreased	76	20.80 (16.57–26.11)	20.57 (1,400.98)	20.36 (16.22)	4.35 (3.69)
Zopiclone	Drug ineffective	65	10.66 (8.21–13.83)	9.41 (495.17)	9.41 (7.25)	3.23 (2.68)
	Insomnia	21	11.73 (7.58–18.16)	11.28 (197.47)	11.28 (7.29)	3.50 (2.31)
	Product quality issue	20	24.32 (15.55–38.05)	23.39 (429.05)	23.37 (14.94)	4.55 (2.86)
	Completed suicide	16	35.45 (21.54–58.35)	34.35 (517.85)	34.31 (20.84)	5.10 (2.82)
	Product substitution issue	13	40.44 (23.30–70.17)	39.42 (486.29)	39.36 (22.68)	5.30 (2.61)
	Anxiety	7	5.60 (2.66–11.81)	5.54 (26.09)	5.54 (2.63)	2.47 (0.79)
	Somnolence	7	4.46 (2.12–9.41)	4.41 (18.54)	4.41 (2.09)	2.14 (0.60)
	Confusional state	7	3.43 (1.62–7.23)	3.39 (11.86)	3.39 (1.61)	1.76 (0.35)
	Fall	7	1.40 (0.67–2.96)	1.40 (0.80)	1.40 (0.66)	0.48 (−0.62)
	Drug effect decreased	7	25.36 (12.02–53.50)	25.02 (161.38)	25.00 (11.85)	4.64 (1.61)

This table lists the top 10 PTs with the highest number of reports (descending by n) associated with nBZRAs, in elderly patients (≥65 years), along with their signal strengths. Values in parentheses represent the lower limit of the 95% confidence interval (for ROR, IC) or the fifth percentile (EBGM05). Signal criteria were: lower limit of ROR, 95% CI > 1, PRR ≥2 and χ^2^ ≥ 4, EBGM05 > 2, and IC025 > 0.

### 3.3 Adverse reaction signal profiles of DORAs

For the DORAs, the adverse event signals for the three drugs revealed a distinct pattern related to sleep/wake regulation mechanisms (Table 3). A prominent commonality was the extremely strong association signals for “Nightmare” and “Abnormal dreams” across all three DORAs. Daridorexant had the strongest signal for “Nightmare” (ROR: 132.78, 95% CI: 97.19–181.39), followed by lemborexant (ROR: 111.81) and suvorexant (ROR: 85.91). Furthermore, lemborexant showed an exceptionally high signal strength for “Sleep paralysis” (ROR: 3528.63), although the absolute number of reports was small (n = 8). Given the small absolute count, this estimate should be interpreted as hypothesis-generating only and its magnitude viewed with caution. Hallucination was also a common signal, significantly present in all three drugs. Regarding common sedation-related adverse reactions, “Somnolence” showed clear signals across all drugs but with moderate strength (ROR: 9.89–13.26). Notably, “Dizziness” showed only a borderline or weak signal in suvorexant (ROR: 1.76, IC025 0.46) and did not rank in the top ten for the other two DORAs. “Fall” did not show a significant signal in lemborexant (ROR: 1.78, IC025–0.34). Signals contrary to the therapeutic intent, such as “Drug ineffective” and “Insomnia,” were detected in all DORAs, but their signal strengths were generally lower than those observed with non-benzodiazepine drugs (e.g., eszopiclone).

**TABLE 3:** Top 10 PTs with signal strengths for adverse events associated with DORAs.

Drug name	PT	n	ROR (95% Cl)	PRR (χ^2^)	EBGM (EBGM05)	IC (IC025)
Lemborexant	Drug ineffective	28	5.44 (3.70–7.98)	5.12 (94.23)	5.12 (3.49)	2.36 (1.61)
	Nightmare	18	111.81 (69.63–179.54)	106.79 (1,880.84)	106.43 (66.28)	6.73 (3.34)
	Somnolence	16	13.26 (8.04–21.86)	12.76 (173.93)	12.76 (7.74)	3.67 (2.20)
	Sleep paralysis	8	3,528.63 (1,689.97–7,367.73)	3,457.55 (24,947.53)	3,120.32 (1,494.42)	11.61 (2.14)
	Seizure	8	19.90 (9.88–40.08)	19.51 (140.59)	19.50 (9.68)	4.29 (1.70)
	Abnormal dreams	7	55.06 (26.06–116.33)	54.10 (364.35)	54.01 (25.56)	5.76 (1.79)
	Hallucination	7	10.69 (5.06–22.58)	10.52 (60.40)	10.52 (4.98)	3.39 (1.23)
	Dyspnoea	7	1.49 (0.71–3.15)	1.48 (1.11)	1.48 (0.70)	0.57 (−0.55)
	Fall	7	1.78 (0.84–3.76)	1.77 (2.35)	1.77 (0.84)	0.82 (−0.34)
	Middle insomnia	6	50.38 (22.48–112.90)	49.64 (285.58)	49.56 (22.12)	5.63 (1.54)
Suvorexant	Drug ineffective	734	12.76 (11.79–13.80)	10.99 (6,727.67)	10.94 (10.12)	3.45 (3.32)
	Nightmare	167	85.91 (73.45–100.48)	83.00 (13,116.33)	80.46 (68.79)	6.33 (5.54)
	Somnolence	158	10.61 (9.06–12.44)	10.30 (1,326.03)	10.27 (8.76)	3.36 (3.04)
	Insomnia	124	7.03 (5.88–8.41)	6.88 (623.59)	6.86 (5.74)	2.78 (2.45)
	Feeling abnormal	109	6.58 (5.44–7.96)	6.46 (503.25)	6.44 (5.33)	2.69 (2.34)
	Abnormal dreams	89	58.21 (47.09–71.95)	57.17 (4,807.38)	55.96 (45.27)	5.81 (4.81)
	Headache	88	2.49 (2.02–3.08)	2.46 (76.99)	2.46 (1.99)	1.30 (0.97)
	Hallucination	87	10.87 (8.79–13.44)	10.69 (762.55)	10.65 (8.61)	3.41 (2.95)
	Wrong technique in product usage process	80	4.47 (3.58–5.58)	4.41 (211.61)	4.41 (3.53)	2.14 (1.76)
	Dizziness	79	1.76 (1.41–2.19)	1.74 (25.23)	1.74 (1.39)	0.80 (0.46)
Daridorexant	Drug ineffective	66	6.53 (5.08–8.41)	6.07 (283.36)	6.07 (4.72)	2.60 (2.13)
	Nightmare	42	132.78 (97.19–181.39)	125.77 (5,160.51)	124.80 (91.36)	6.96 (4.55)
	Headache	33	5.90 (4.16–8.36)	5.70 (128.64)	5.69 (4.02)	2.51 (1.82)
	Fatigue	32	3.35 (2.35–4.77)	3.25 (50.59)	3.25 (2.28)	1.70 (1.09)
	Insomnia	30	10.61 (7.37–15.28)	10.25 (251.06)	10.24 (7.11)	3.36 (2.45)
	Somnolence	24	9.89 (6.59–14.85)	9.62 (185.89)	9.62 (6.40)	3.27 (2.25)
	Hallucination	20	15.48 (9.93–24.14)	15.12 (263.84)	15.10 (9.69)	3.92 (2.54)
	Feeling abnormal	17	6.31 (3.90–10.21)	6.20 (74.37)	6.20 (3.83)	2.63 (1.58)
	Nausea	17	1.85 (1.14–2.99)	1.83 (6.48)	1.83 (1.13)	0.87 (0.12)
	Wrong technique in product usage process	15	5.18 (3.11–8.63)	5.10 (49.58)	5.10 (3.06)	2.35 (1.29)

This table lists the top 10 PTs with the highest number of reports (descending by n) associated with three DORAs, in elderly patients (≥65 years), along with their signal strengths. Values in parentheses represent the lower limit of the 95% confidence interval (for ROR, IC) or the fifth percentile (EBGM05). Signal criteria were: lower limit of ROR, 95% CI > 1, PRR ≥2 and χ^2^ ≥ 4, EBGM05 > 2, and IC025 > 0.

### 3.4 Heatmap analysis of adverse reaction signals across drugs based on RORs

**FIGURE 4:**
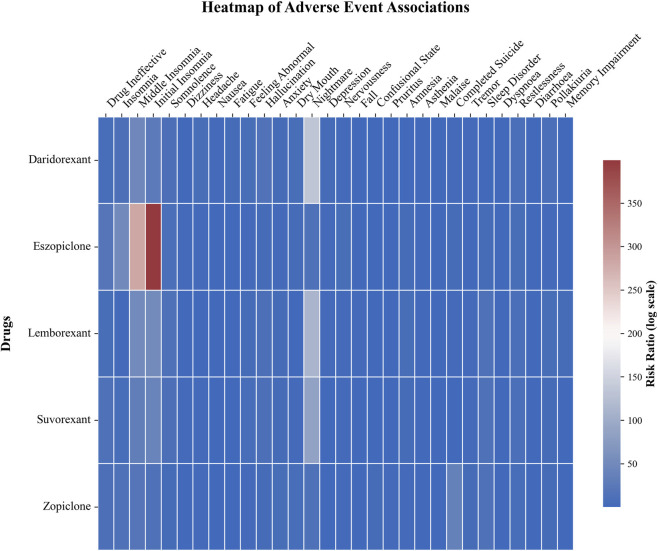
Heatmap of RORs for the top 30 PTs of adverse events across five drugs. This heatmap provides a visual comparison of the association strengths, represented by the natural logarithm of the Reporting Odds Ratio [ln(ROR)], between the five study drugs and the top 30 most frequently reported PTs of adverse events. Color intensity indicates signal strength, ranging from blue [weaker or no association, lower ln(ROR)] to red [stronger association, higher ln(ROR)]. White cells indicate that the drug-event combination was not among the top 30 for that drug or data were missing.

The heatmap generated based on ROR displayed disparities in the top 30 adverse event signals among the five drugs. Among the nBZRAs, eszopiclone showed the highest ROR values for “Initial Insomnia” and “Middle Insomnia.” Signals for dual orexin receptor antagonists were concentrated in “Nightmare”, “Abnormal Dreams”, and “Sleep Paralysis” with lemborexant exhibiting the highest ROR value for “Sleep Paralysis” (Figure 4). The specific values of the four disproportionality analyses corresponding to the top 30 adverse events ranked by report count for the five drugs are detailed in Supplementary Table S3.

### 3.5 Spectra of adverse events revealed by multiple positive signals

As outlined in Supplementary Table S4 (listing the top 20 Primary Terms positive across all four statistical methods), for nBZRAs, the common predominant events for eszopiclone and zopiclone were “Drug Ineffective” and “Insomnia”. Specific events for eszopiclone included “Dysgeusia” (Report Count: 598) and CNS events such as “Hallucination”, “Delirium”, and “Amnesia”. Reports for zopiclone included “Completed Suicide”, “Anxiety”, “Dementia”, and “Orthostatic Hypotension”.

Within the dual orexin receptor antagonist class, the three drugs shared reports of “Drug Ineffective” and “Nightmare,” with “Nightmare” ranking second for lemborexant and first for daridorexant. Reports commonly included events like “Sleep Paralysis,” “Abnormal Dreams,” and “Hallucination”. Suvorexant and daridorexant also showed frequent reports related to product use processes. The list for lemborexant included events such as “Seizure,” “Aspiration Pneumonia,” and “Interstitial Lung Disease”.

### 3.6 Analysis of signals for fall-related adverse events

The signal detection results for fall-related events from the FAERS database are shown in Table 4. The point estimates of the ROR were 1.40 for zopiclone and 1.78 for lemborexant; however, their 95% CIs both included 1, indicating no statistical significance. Eszopiclone showed an ROR of 0.49 (95% CI: 0.35–0.69), with the upper limit of its CI below 1, suggesting a low association signal. For suvorexant (ROR = 0.90, 95% CI: 0.67–1.22) and daridorexant (ROR = 0.76, 95% CI: 0.34–1.69), the ROR point estimates were below 1, and their CIs included 1, indicating no significant signals were detected. The signal strength analyses using PRR, EBGM, and IC yielded results consistent with the trends observed in the ROR analysis.

**TABLE 4:** Signal detection results for fall-related adverse events associated with five drugs.

Fall	n	ROR (95%Cl)	PRR (χ^2^)	EBGM (EBGM05)	IC (IC025)
Eszopiclone	33	0.49 (0.35–0.69)	0.49 (17.28)	0.49 (0.35)	−1.02 (−1.49)
Zopiclone	7	1.40 (0.67–2.96)	1.40 (0.80)	1.40 (0.66)	0.48 (−0.62)
Lemborexant	7	1.78 (0.84–3.76)	1.77 (2.35)	1.77 (0.84)	0.82 (−0.34)
Suvorexant	44	0.90 (0.67–1.22)	0.90 (0.45)	0.90 (0.67)	−0.14 (−0.57)
Daridorexant	6	0.76 (0.34–1.69)	0.76 (0.46)	0.76 (0.34)	−0.39 (−1.44)

n: Number of cases reporting the drug-fall event combination.

### 3.7 Distribution of adverse events by SOCs

**FIGURE 5:**
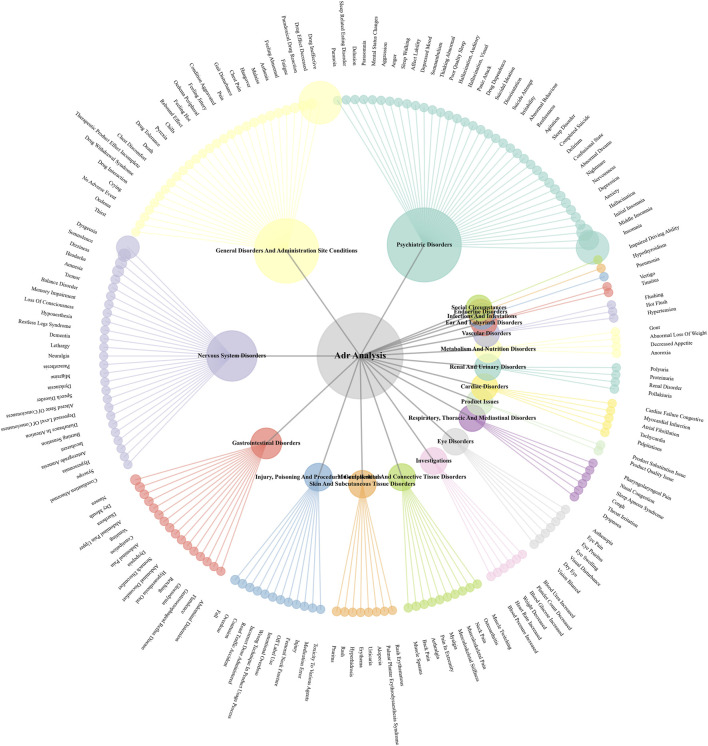
The PT hierarchical mapping of adverse events for five drugs.

At the System Organ Class level, the distribution of adverse events further corroborated the findings at the PT level (Supplementary Table S5). Overall, the vast majority of reports were concentrated in three SOCs: “Psychiatric disorders,” “General disorders and administration site conditions,” and “Nervous system disorders.” Specifically, eszopiclone had the highest number of reports in the “Psychiatric disorders” and “Nervous system disorders” SOCs. Among the DORAs, suvorexant also had the highest report counts in the three aforementioned primary SOCs. Notably, zopiclone showed a relatively higher proportion of reports in the “Injury, poisoning and procedural complications” and “Product issues” SOCs relative to its total report volume. As shown in Figure 5, the PT hierarchical mapping of adverse events for five drugs reveals the organ system distribution characteristics.

## 4 Discussion

This study, through a systematic analysis of the FAERS database, provides an initial delineation of adverse event reporting profiles associated with the use of nBZRAs and DORAs in elderly patients. The analytical results indicate differences in the signal spectra between the two drug classes. For nBZRAs, particularly eszopiclone, a combination of high-strength signals was observed centering on “Drug ineffective” and specific insomnia subtypes (e.g., “Initial insomnia,” “Middle insomnia”), alongside a prominent signal for “Dysgeusia”. In contrast, DORAs as a class displayed a distinct set of signals, with generally higher signal strengths for events related to abnormal sleep experiences (e.g., “Nightmare,” “Abnormal dreams”) and perceptual changes (e.g., “Hallucination”). A notable observation is that neither drug class generated a statistically significant signal for “Fall” within the context of this dataset and under our analytical methods. These observed differences in signals suggest the possibility of distinct safety profiles for these two classes of insomnia medications in the elderly population. It is important to emphasize that disproportionality analyses identify statistical associations between drugs and reported adverse events; they do not establish causality. The signals presented here should be regarded as hypothesis generating and require confirmation in controlled epidemiological studies.

Building upon the established yet often misaligned risk-benefit profiles of hypnotics, our analysis provides critical real-world safety data for older adults—a key gap in knowledge translation. While quantitative analyses indicate that nBZRAs carry a higher adverse event burden compared to the more favorable efficacy-tolerability balance of DORAs (Cheung et al., 2024), clinical practice often does not reflect this. For instance, nBZRA prescriptions increased significantly in China from 2015 to 2019 despite their known risks (Lou et al., 2022). This underscores the imperative for post-marketing surveillance specifically in the elderly, a demographic with high hypnotic use and heightened vulnerability. Our comparative pharmacovigilance study of FAERS data directly addresses this need.

The well-documented safety concerns of traditional benzodiazepines and nBZRAs in geriatrics provide critical context. These include misuse potential and risks of physical harm. Survey data show that while population-level nBZRA misuse is lower, user-level misuse is notable, and benzodiazepine misuse is strongly linked to severe mental health outcomes and polydrug use (McHugh et al., 2023). Clinically, increased fall risk is a major concern for older adults (Capiau et al., 2023), and systematic reviews also associate these drugs with elevated venous thromboembolism risk (Yang et al., 2025). Our findings on subjective sleep-related adverse events should be considered alongside objective evidence of functional impairment. A recent network meta-analysis of randomized controlled trials demonstrated that both zopiclone and DORAs are associated with residual next-morning driving performance deficits, although the magnitude varies by drug and dose (Fornaro et al., 2024b). However, a recent cohort study of elderly drivers in collisions found no significant link between these hypnotics and severe injury hospitalization (Benhamed et al., 2025). This aligns with our pharmacovigilance finding of no significant disproportionality signal for “Falls.” This suggests that while fall risk remains a paramount clinical driver for caution, it may not be disproportionately reported in spontaneous systems, possibly due to under-reporting or multifactorial etiology in the elderly. Overall, the literature confirms a complex and often unfavorable safety profile for BZRAs/nBZRAs in this population.

For nBZRAs, our signals of “Drug ineffective” and “Dysgeusia” (notably for eszopiclone) align with broader evidence. A large network meta-analysis confirms nBZRA efficacy for sleep but also a higher adverse event risk versus placebo (Pan et al., 2023). Of note, the adverse event terms “Drug ineffective” and “Insomnia” (and related insomnia subtypes) are included in the primary signal tables. These terms are coded as adverse events in FAERS and reflect patient-reported therapeutic failure or dissatisfaction. They do not represent classical adverse drug reactions (e.g., toxicity or hypersensitivity), and their signal strengths should be interpreted as indicators of perceived lack of efficacy rather than harm. To maintain transparency and avoid selective reporting, we present all reported events in the main tables. Furthermore, U.S. overdose death data reveal a significant increase in fatalities involving non-benzodiazepine sedative-hypnotics, especially with opioids or benzodiazepines (Tardelli et al., 2022). Although our analysis did not show significant fatal outcome signals—potentially due to reporting or methodological factors—this mortality data underscores their lethal potential in scenarios of misuse or polypharmacy. Thus, nBZRAs present a profile of efficacy coupled with a tangible burden of adverse effects and severe risks in specific contexts. To be noted, the signal for “Completed suicide” with zopiclone warrants cautious interpretation. Disproportionality alone does not imply causation; this association is likely confounded by underlying psychiatric comorbidities, concomitant use of psychotropic medications, and the indication itself. Our findings should be viewed as hypothesis-generating, and further research using controlled designs is necessary to disentangle drug-attributable risk from confounding factors.

Regarding DORAs, our stronger signals for “Nightmare” and “Hallucination” are mechanistically consistent. DORAs work by blocking orexin receptors, which stabilize wakefulness. This inhibition can disrupt sleep-wake state boundaries, leading to intrusion of REM-sleep phenomena like vivid dreams or hallucinations. This mechanism explains why systematic reviews and other pharmacovigilance studies consistently report somnolence, abnormal dreams, and sleep paralysis as characteristic adverse events for DORAs (Jiang et al., 2024; Sun et al., 2025; Xue et al., 2022; Żełabowski et al., 2025). Variations in signal ranking between studies (e.g., “sleep paralysis” vs. “nightmare” as top signals) may stem from methodological or population differences. Convergent evidence solidifies that DORAs have a distinct mechanism with a recognizable safety signature centered on sleep-wake dysregulation.

This evolving understanding of DORA safety informs their potential application in complex clinical scenarios, or “special populations”. Beyond our general elderly focus, evidence suggests DORAs may be advantageous where GABAergic drugs are problematic. For instance, in patients with obstructive sleep apnea (OSA), a condition prevalent in the elderly, DORAs have been shown to improve sleep without exacerbating respiratory events, a critical safety consideration where sedatives are typically used with caution (Yeh et al., 2025). Furthermore, exploratory research indicates potential therapeutic roles for DORAs beyond primary insomnia, including in the modulation of sleep-epilepsy interactions and as a symptomatic treatment for sleep disturbances in Alzheimer’s disease, with some preclinical hints at disease-modifying potential (Berteotti et al., 2023; Carpi et al., 2024). Early data support their use in insomnia with psychiatric comorbidity without worsening symptoms (Kishi et al., 2024). However, vigilance for their characteristic effects, such as narcolepsy-like symptoms (Na et al., 2024), is crucial in these groups. Conversely, benzodiazepines in critical care are linked to poor sleep outcomes (van der Hoeven et al., 2024), highlighting their limitations. Therefore, while DORAs present a mechanistically distinct and often favorable option for insomnia in several special populations, their safety profile-particularly regarding sleep-wake state disturbances-must be carefully weighed against the specific risks and alternatives in each clinical context.

These safety profiles inform practical clinical management. Treatment selection should be guided by efficacy, safety, and the principle of individualized therapy. For DORAs, network meta-analyses support their efficacy, with specific agents and doses showing advantages for different sleep parameters (e.g., lemborexant for sleep onset, daridorexant for sleep maintenance) (Kishi et al., 2025; Xue et al., 2023), alongside their characteristic side effects requiring monitoring (e.g., abnormal sleep experiences, somnolence) (Xue et al., 2023). Optimization extends beyond drug choice to include administration timing, as proper scheduling of hypnotics and other medications affecting sleep-wake cycles can enhance efficacy and minimize daytime sequelae (Khoshnevis et al., 2023). Regarding treatment duration, expert consensus opposes a blanket prohibition on long-term use for certain agents, including DORAs and some nBZRAs, but underscores the necessity of periodic re-evaluation of the risk-benefit balance, particularly in older adults (Zee et al., 2023). When deprescribing, gradual tapering is recommended, and switching to agents like daridorexant may facilitate the process due to lower rebound risk (Palagini et al., 2025). Furthermore, non-pharmacological interventions, such as CBT-I, remain first-line, and emerging modalities like acupuncture show mechanistic promise in preclinical models (Palagini et al., 2025; Zhang et al., 2023).

This study possesses several notable strengths. Firstly, it leverages the large-scale FAERS database, spanning over 20 years (2004–2025) and including 5,447 adverse event reports from elderly patients, which provides substantial statistical power and representativeness. Secondly, the study employs a comprehensive disproportionality analysis approach (ROR, PRR, IC, EBGM) with stringent thresholds, effectively minimizing false-positive signals and enhancing the robustness of signal detection. Furthermore, the research specifically focuses on the elderly population (≥65 years), a group with high rates of polypharmacy and comorbidities, making the findings clinically relevant and significant for public health. Lastly, this is one of the first real-world studies to systematically compare the safety profiles of nBZRAs and DORAs, addressing an important evidence gap. The consistency between the detected signals and known pharmacological mechanisms (e.g., dream-related events with DORAs’ action on sleep-wake regulation) further strengthens the interpretability of the results. Our pharmacovigilance findings reveal persistent “drug ineffective” signals for nBZRAs, warranting periodic efficacy reassessment; eszopiclone shows strong dysgeusia signals, meriting routine inquiry. DORAs are linked to nightmares and hallucinations, supporting pretreatment counseling. Fall signals are not significant but clinical vigilance remains prudent. These profiles inform individualized prescribing: DORAs suit nBZRA-intolerant patients or those with long-term efficacy concerns, while nBZRAs may be preferred in those vulnerable to neuropsychiatric events. Signals should be triangulated with trials and guidelines to guide shared decisions.

This study has several limitations. First, as a spontaneous reporting database, FAERS is subject to biases (under-reporting, channeling, notoriety) and cannot establish causality; reporter and geographic variations may affect signal comparability, and no stratified analyses were performed. Second, our nBZRA analysis was confined to eszopiclone and zopiclone due to low counts or off-label use of others, limiting class-level generalizability despite enhanced internal validity. Third, fall-related events were assessed using only the PT “Fall”, potentially missing related outcomes (e.g., fracture, syncope); thus, the null finding does not rule out disproportionate reporting for fall injuries. Fourth, some signals (e.g., sleep paralysis for lemborexant) were based on <10 cases; despite Bayesian stabilization, these estimates remain unstable, and absence of detailed clinical data precludes confounding control. Fifth, FAERS lacks exposure denominators, precluding incidence estimates; disproportionality reflects relative reporting, not causal risk, and is influenced by non-risk factors. No subgroup or sensitivity analyses were conducted, and underreporting (e.g., falls) is likely. Triangulation with evidence from other study designs (e.g., cohort studies, registries, meta-analyses of RCTs) is essential before clinical conclusions.

## 5 Conclusion

This study demonstrates that nBZRAs and DORAs exhibit distinct safety profiles in the elderly: nBZRAs are primarily associated with therapeutic failure and dysgeusia, whereas DORAs show stronger signals for neuropsychiatric events such as nightmare and hallucination. These findings provide real-world evidence to support individualized treatment selection for insomnia in older adults, highlighting the need to balance efficacy with specific risks. The study also underscores the value of large-scale pharmacovigilance data in differentiating post-marketing risk profiles, offering clear implications for enhancing medication safety in the aging population.

## Data Availability

The original contributions presented in the study are included in the article/Supplementary Material, further inquiries can be directed to the corresponding author.
