# Supplementary material for: Comparative pharmacovigilance of non-benzodiazepine receptor agonists versus dual orexin receptor antagonists for insomnia in older adults
Source: Front Pharmacol. 2026 Mar 10;17:1788736. doi: 10.3389/fphar.2026.1788736 (PMC13008954; doi:10.3389/fphar.2026.1788736)
Supplement: Supplementary file 1 [file Table1.docx]

Supplementary Material

# Supplementary Figures and Tables

**Supplementary TABLE 1.** Age and sex distribution of adverse event reports across drug groups

| **Age** | **Sex** | **Eszopiclone** | **Zopiclone** | **Lemborexant** | **Suvorexant** | **Daridorexant** |
| --- | --- | --- | --- | --- | --- | --- |
| 60-74 | F | 828 | 58 | 50 | 533 | 92 |
| 60-74 | M | 541 | 41 | 42 | 367 | 63 |
| 60-74 | Missing | 15 | 3 | 0 | 12 | 0 |
| 75-89 | F | 782 | 39 | 36 | 518 | 54 |
| 75-89 | M | 581 | 22 | 27 | 394 | 43 |
| 75-89 | Missing | 8 | 0 | 1 | 10 | 1 |
| 90+ | F | 54 | 5 | 13 | 93 | 5 |
| 90+ | M | 39 | 2 | 7 | 59 | 4 |
| 90+ | Missing | 1 | 0 | 0 | 3 | 1 |

**Note:** Data are sourced from the FAERS database (2004Q1–2025Q2), including only patients aged ≥65 years. The grouped bar chart displays the number of reports for males and females within different age strata (65-74, 75-89, ≥90 years) for each drug group. F: female, M: male, 90+: ≥90 years.

**Supplementary TABLE 2.** Temporal trends in the quarterly number of adverse event reports for each drug

.

| **Year** | **Eszopiclone** | **Zopiclone** | **Lemborexant** | **Suvorexant** | **Daridorexant** |
| --- | --- | --- | --- | --- | --- |
| 2005 | 556 | - | - | - | - |
| 2006 | 1186 | - | - | - | - |
| 2007 | 721 | - | - | - | - |
| 2008 | 10 | 7 | - | - | - |
| 2009 | 9 | 2 | - | - | - |
| 2010 | 11 | 3 | - | - | - |
| 2011 | 9 | 2 | - | - | - |
| 2012 | 14 | 2 | - | - | - |
| 2013 | 14 | 4 | - | - | - |
| 2014 | 19 | 14 | - | 1 | - |
| 2015 | 20 | 10 | - | 338 | - |
| 2016 | 19 | 10 | - | 421 | - |
| 2017 | 31 | 16 | - | 338 | - |
| 2018 | 32 | 13 | - | 212 | - |
| 2019 | 33 | 14 | - | 172 | - |
| 2020 | 30 | 13 | 36 | 121 | - |
| 2021 | 18 | 10 | 43 | 98 | - |
| 2022 | 31 | 19 | 34 | 83 | 15 |
| 2023 | 51 | 17 | 25 | 96 | 180 |
| 2024 | 31 | 12 | 24 | 69 | 36 |
| 2025 | 4 | 2 | 14 | 40 | 32 |

**Supplementary TABLE 3.** The specific values of the four disproportionality analyses corresponding to the top 30 adverse events ranked by report count for the five drugs

| **Drug Name** | **PT** | **n** | **ROR(95%Cl)** | **PRR(χ²)** | **EBGM(EBGM05)** | **IC(IC025)** |
| --- | --- | --- | --- | --- | --- | --- |
| **Eszopiclone** | Drug ineffective | 1415 | 19.36 ( 18.26 - 20.54 ) | 15.48 ( 19276.45 ) | 15.36 ( 14.48) | 3.94 ( 3.84 ) |
|  | Insomnia | 1009 | 48.73 ( 45.54 - 52.13 ) | 41.53 ( 39199.56 ) | 40.66 ( 38.00) | 5.35 ( 5.19 ) |
|  | Dysgeusia | 598 | 74.86 ( 68.73 - 81.53 ) | 68.26 ( 38305.32 ) | 65.92 ( 60.53) | 6.04 ( 5.77 ) |
|  | Middle insomnia | 471 | 282.48 ( 255.72 - 312.04 ) | 262.67 ( 107874.78 ) | 230.84 ( 208.97) | 7.85 ( 7.13 ) |
|  | Initial insomnia | 313 | 399.43 ( 352.90 - 452.10 ) | 380.80 ( 98754.52 ) | 317.29 ( 280.33) | 8.31 ( 7.12 ) |
|  | Somnolence | 172 | 8.36 ( 7.18 - 9.73 ) | 8.17 ( 1080.69 ) | 8.14 ( 6.99) | 3.02 ( 2.74 ) |
|  | Dizziness | 119 | 1.93 ( 1.61 - 2.31 ) | 1.91 ( 52.20 ) | 1.91 ( 1.59) | 0.93 ( 0.66 ) |
|  | Headache | 113 | 2.33 ( 1.93 - 2.80 ) | 2.30 ( 83.87 ) | 2.30 ( 1.91) | 1.20 ( 0.91 ) |
|  | Nausea | 77 | 0.98 ( 0.78 - 1.22 ) | 0.98 ( 0.04 ) | 0.98 ( 0.78) | -0.03 ( -0.36 ) |
|  | Drug effect decreased | 76 | 20.80 ( 16.57 - 26.11 ) | 20.57 ( 1400.98 ) | 20.36 ( 16.22) | 4.35 ( 3.69 ) |
|  | Paradoxical drug reaction | 59 | 233.74 ( 178.19 - 306.61 ) | 231.69 ( 12077.03 ) | 206.57 ( 157.48) | 7.69 ( 5.15 ) |
|  | Fatigue | 49 | 0.59 ( 0.44 - 0.77 ) | 0.59 ( 14.31 ) | 0.59 ( 0.44) | -0.77 ( -1.16 ) |
|  | Feeling abnormal | 44 | 1.90 ( 1.41 - 2.56 ) | 1.89 ( 18.64 ) | 1.89 ( 1.41) | 0.92 ( 0.46 ) |
|  | Hallucination | 42 | 3.77 ( 2.78 - 5.10 ) | 3.75 ( 84.68 ) | 3.74 ( 2.76) | 1.90 ( 1.37 ) |
|  | Anxiety | 40 | 2.38 ( 1.75 - 3.25 ) | 2.38 ( 31.90 ) | 2.37 ( 1.74) | 1.25 ( 0.75 ) |
|  | Dry mouth | 40 | 3.98 ( 2.91 - 5.43 ) | 3.96 ( 88.35 ) | 3.95 ( 2.89) | 1.98 ( 1.43 ) |
|  | Nightmare | 36 | 12.77 ( 9.19 - 17.74 ) | 12.71 ( 385.90 ) | 12.63 ( 9.09) | 3.66 ( 2.79 ) |
|  | Depression | 36 | 2.68 ( 1.93 - 3.72 ) | 2.67 ( 37.75 ) | 2.67 ( 1.92) | 1.42 ( 0.88 ) |
|  | Nervousness | 36 | 6.06 ( 4.37 - 8.41 ) | 6.03 ( 150.84 ) | 6.02 ( 4.33) | 2.59 ( 1.93 ) |
|  | Abnormal dreams | 34 | 15.76 ( 11.24 - 22.11 ) | 15.69 ( 463.82 ) | 15.57 ( 11.10) | 3.96 ( 2.97 ) |
|  | Fall | 33 | 0.49 ( 0.35 - 0.69 ) | 0.49 ( 17.28 ) | 0.49 ( 0.35) | -1.02 ( -1.49 ) |
|  | Confusional state | 33 | 1.20 ( 0.85 - 1.69 ) | 1.20 ( 1.10 ) | 1.20 ( 0.85) | 0.26 ( -0.24 ) |
|  | Delirium | 32 | 4.72 ( 3.33 - 6.68 ) | 4.70 ( 93.06 ) | 4.69 ( 3.31) | 2.23 ( 1.57 ) |
|  | Pruritus | 28 | 0.76 ( 0.52 - 1.10 ) | 0.76 ( 2.14 ) | 0.76 ( 0.52) | -0.40 ( -0.92 ) |
|  | Amnesia | 28 | 4.49 ( 3.10 - 6.51 ) | 4.47 ( 75.41 ) | 4.47 ( 3.08) | 2.16 ( 1.46 ) |
|  | Asthenia | 27 | 0.46 ( 0.31 - 0.67 ) | 0.46 ( 17.22 ) | 0.46 ( 0.32) | -1.12 ( -1.64 ) |
|  | Malaise | 26 | 0.50 ( 0.34 - 0.74 ) | 0.51 ( 12.70 ) | 0.51 ( 0.34) | -0.98 ( -1.51 ) |
|  | Completed suicide | 22 | 3.55 ( 2.34 - 5.40 ) | 3.54 ( 40.13 ) | 3.54 ( 2.33) | 1.82 ( 1.07 ) |
|  | Tremor | 21 | 1.09 ( 0.71 - 1.67 ) | 1.09 ( 0.15 ) | 1.09 ( 0.71) | 0.12 ( -0.50 ) |
|  | Sleep disorder | 20 | 3.40 ( 2.19 - 5.27 ) | 3.39 ( 33.69 ) | 3.39 ( 2.18) | 1.76 ( 0.97 ) |
| **Zopiclone** | Drug ineffective | 65 | 10.66 ( 8.21 - 13.83 ) | 9.41 ( 495.17 ) | 9.41 ( 7.25) | 3.23 ( 2.68 ) |
|  | Insomnia | 21 | 11.73 ( 7.58 - 18.16 ) | 11.28 ( 197.47 ) | 11.28 ( 7.29) | 3.50 ( 2.31 ) |
|  | Product quality issue | 20 | 24.32 ( 15.55 - 38.05 ) | 23.39 ( 429.05 ) | 23.37 ( 14.94) | 4.55 ( 2.86 ) |
|  | Completed suicide | 16 | 35.45 ( 21.54 - 58.35 ) | 34.35 ( 517.85 ) | 34.31 ( 20.84) | 5.10 ( 2.82 ) |
|  | Product substitution issue | 13 | 40.44 ( 23.30 - 70.17 ) | 39.42 ( 486.29 ) | 39.36 ( 22.68) | 5.30 ( 2.61 ) |
|  | Anxiety | 7 | 5.60 ( 2.66 - 11.81 ) | 5.54 ( 26.09 ) | 5.54 ( 2.63) | 2.47 ( 0.79 ) |
|  | Somnolence | 7 | 4.46 ( 2.12 - 9.41 ) | 4.41 ( 18.54 ) | 4.41 ( 2.09) | 2.14 ( 0.60 ) |
|  | Confusional state | 7 | 3.43 ( 1.62 - 7.23 ) | 3.39 ( 11.86 ) | 3.39 ( 1.61) | 1.76 ( 0.35 ) |
|  | Fall | 7 | 1.40 ( 0.67 - 2.96 ) | 1.40 ( 0.80 ) | 1.40 ( 0.66) | 0.48 ( -0.62 ) |
|  | Drug effect decreased | 7 | 25.36 ( 12.02 - 53.50 ) | 25.02 ( 161.38 ) | 25.00 ( 11.85) | 4.64 ( 1.61 ) |
|  | Headache | 7 | 1.91 ( 0.91 - 4.03 ) | 1.90 ( 3.01 ) | 1.90 ( 0.90) | 0.93 ( -0.26 ) |
|  | Dysgeusia | 6 | 8.91 ( 3.98 - 19.94 ) | 8.82 ( 41.63 ) | 8.82 ( 3.94) | 3.14 ( 0.96 ) |
|  | Dizziness | 6 | 1.29 ( 0.58 - 2.88 ) | 1.28 ( 0.38 ) | 1.28 ( 0.57) | 0.36 ( -0.80 ) |
|  | Sleep disorder | 6 | 13.70 ( 6.12 - 30.65 ) | 13.55 ( 69.76 ) | 13.54 ( 6.05) | 3.76 ( 1.18 ) |
|  | Pruritus | 5 | 1.82 ( 0.75 - 4.38 ) | 1.81 ( 1.82 ) | 1.81 ( 0.75) | 0.85 ( -0.51 ) |
|  | Dementia | 5 | 10.86 ( 4.50 - 26.21 ) | 10.76 ( 44.29 ) | 10.76 ( 4.46) | 3.43 ( 0.85 ) |
|  | Tremor | 5 | 3.47 ( 1.44 - 8.38 ) | 3.45 ( 8.72 ) | 3.45 ( 1.43) | 1.79 ( 0.11 ) |
|  | Death | 5 | 0.48 ( 0.20 - 1.16 ) | 0.49 ( 2.79 ) | 0.49 ( 0.20) | -1.04 ( -2.10 ) |
|  | Dry mouth | 5 | 6.64 ( 2.75 - 16.03 ) | 6.58 ( 23.71 ) | 6.58 ( 2.73) | 2.72 ( 0.58 ) |
|  | Fatigue | 4 | 0.64 ( 0.24 - 1.70 ) | 0.64 ( 0.82 ) | 0.64 ( 0.24) | -0.64 ( -1.84 ) |
|  | Hallucination | 4 | 4.78 ( 1.79 - 12.80 ) | 4.75 ( 11.88 ) | 4.75 ( 1.78) | 2.25 ( 0.14 ) |
|  | Wrong technique in product usage process | 4 | 2.15 ( 0.80 - 5.75 ) | 2.14 ( 2.43 ) | 2.14 ( 0.80) | 1.10 ( -0.50 ) |
|  | Abnormal loss of weight | 4 | 54.35 ( 20.30 - 145.54 ) | 53.93 ( 207.38 ) | 53.82 ( 20.10) | 5.75 ( 0.92 ) |
|  | Rash | 4 | 1.30 ( 0.49 - 3.48 ) | 1.30 ( 0.27 ) | 1.30 ( 0.48) | 0.38 ( -1.01 ) |
|  | Feeling abnormal | 4 | 2.31 ( 0.86 - 6.17 ) | 2.30 ( 2.93 ) | 2.30 ( 0.86) | 1.20 ( -0.43 ) |
|  | Nausea | 3 | 0.51 ( 0.16 - 1.57 ) | 0.51 ( 1.44 ) | 0.51 ( 0.16) | -0.98 ( -2.24 ) |
|  | Middle insomnia | 3 | 19.72 ( 6.34 - 61.39 ) | 19.61 ( 52.96 ) | 19.60 ( 6.30) | 4.29 ( 0.34 ) |
|  | Pyrexia | 3 | 1.07 ( 0.34 - 3.32 ) | 1.07 ( 0.01 ) | 1.07 ( 0.34) | 0.09 ( -1.38 ) |
|  | Hypertension | 3 | 1.48 ( 0.48 - 4.61 ) | 1.48 ( 0.47 ) | 1.48 ( 0.48) | 0.56 ( -1.05 ) |
|  | Condition aggravated | 3 | 1.40 ( 0.45 - 4.35 ) | 1.40 ( 0.34 ) | 1.40 ( 0.45) | 0.48 ( -1.11 ) |
| **Lemborexant** | Drug ineffective | 28 | 5.44 ( 3.70 - 7.98 ) | 5.12 ( 94.23 ) | 5.12 ( 3.49) | 2.36 ( 1.61 ) |
|  | Nightmare | 18 | 111.81 ( 69.63 - 179.54 ) | 106.79 ( 1880.84 ) | 106.43 ( 66.28) | 6.73 ( 3.34 ) |
|  | Somnolence | 16 | 13.26 ( 8.04 - 21.86 ) | 12.76 ( 173.93 ) | 12.76 ( 7.74) | 3.67 ( 2.20 ) |
|  | Sleep paralysis | 8 | 3528.63 ( 1689.97 - 7367.73 ) | 3457.55 ( 24947.53 ) | 3120.32 ( 1494.42) | 11.61 ( 2.14 ) |
|  | Seizure | 8 | 19.90 ( 9.88 - 40.08 ) | 19.51 ( 140.59 ) | 19.50 ( 9.68) | 4.29 ( 1.70 ) |
|  | Abnormal dreams | 7 | 55.06 ( 26.06 - 116.33 ) | 54.10 ( 364.35 ) | 54.01 ( 25.56) | 5.76 ( 1.79 ) |
|  | Hallucination | 7 | 10.69 ( 5.06 - 22.58 ) | 10.52 ( 60.40 ) | 10.52 ( 4.98) | 3.39 ( 1.23 ) |
|  | Dyspnoea | 7 | 1.49 ( 0.71 - 3.15 ) | 1.48 ( 1.11 ) | 1.48 ( 0.70) | 0.57 ( -0.55 ) |
|  | Fall | 7 | 1.78 ( 0.84 - 3.76 ) | 1.77 ( 2.35 ) | 1.77 ( 0.84) | 0.82 ( -0.34 ) |
|  | Middle insomnia | 6 | 50.38 ( 22.48 - 112.90 ) | 49.64 ( 285.58 ) | 49.56 ( 22.12) | 5.63 ( 1.54 ) |
|  | Feeling abnormal | 6 | 4.40 ( 1.97 - 9.87 ) | 4.35 ( 15.55 ) | 4.35 ( 1.94) | 2.12 ( 0.46 ) |
|  | Dizziness | 6 | 1.63 ( 0.73 - 3.66 ) | 1.62 ( 1.45 ) | 1.62 ( 0.73) | 0.70 ( -0.53 ) |
|  | Altered state of consciousness | 6 | 27.97 ( 12.49 - 62.66 ) | 27.56 ( 153.55 ) | 27.54 ( 12.29) | 4.78 ( 1.42 ) |
|  | Delirium | 6 | 15.04 ( 6.71 - 33.69 ) | 14.83 ( 77.42 ) | 14.82 ( 6.62) | 3.89 ( 1.22 ) |
|  | Nausea | 5 | 1.07 ( 0.44 - 2.59 ) | 1.07 ( 0.02 ) | 1.07 ( 0.44) | 0.10 ( -1.11 ) |
|  | Pneumonia aspiration | 5 | 16.82 ( 6.96 - 40.66 ) | 16.62 ( 73.44 ) | 16.62 ( 6.88) | 4.05 ( 1.02 ) |
|  | Fatigue | 5 | 1.01 ( 0.42 - 2.44 ) | 1.01 ( 0.00 ) | 1.01 ( 0.42) | 0.02 ( -1.18 ) |
|  | Interstitial lung disease | 5 | 7.64 ( 3.16 - 18.46 ) | 7.55 ( 28.47 ) | 7.55 ( 3.13) | 2.92 ( 0.66 ) |
|  | Drug interaction | 5 | 3.35 ( 1.39 - 8.09 ) | 3.32 ( 8.12 ) | 3.32 ( 1.37) | 1.73 ( 0.07 ) |
|  | Insomnia | 4 | 2.73 ( 1.02 - 7.32 ) | 2.72 ( 4.36 ) | 2.72 ( 1.01) | 1.44 ( -0.28 ) |
|  | Sleep terror | 4 | 331.35 ( 123.13 - 891.71 ) | 328.02 ( 1290.91 ) | 324.70 ( 120.66) | 8.34 ( 1.00 ) |
|  | Intentional overdose | 4 | 33.53 ( 12.52 - 89.83 ) | 33.20 ( 124.84 ) | 33.17 ( 12.38) | 5.05 ( 0.86 ) |
|  | Asthenia | 4 | 1.15 ( 0.43 - 3.08 ) | 1.15 ( 0.08 ) | 1.15 ( 0.43) | 0.20 ( -1.14 ) |
|  | Oxygen saturation decreased | 4 | 7.73 ( 2.89 - 20.70 ) | 7.66 ( 23.19 ) | 7.66 ( 2.86) | 2.94 ( 0.42 ) |
|  | Dysphagia | 4 | 5.31 ( 1.98 - 14.21 ) | 5.26 ( 13.83 ) | 5.26 ( 1.96) | 2.40 ( 0.21 ) |
|  | Anxiety | 4 | 4.03 ( 1.51 - 10.80 ) | 4.00 ( 9.03 ) | 4.00 ( 1.49) | 2.00 ( 0.02 ) |
|  | Headache | 4 | 1.38 ( 0.51 - 3.69 ) | 1.37 ( 0.41 ) | 1.37 ( 0.51) | 0.46 ( -0.95 ) |
|  | Sleep disorder | 4 | 11.53 ( 4.30 - 30.87 ) | 11.42 ( 38.05 ) | 11.42 ( 4.26) | 3.51 ( 0.59 ) |
|  | Suicide attempt | 4 | 36.56 ( 13.64 - 97.95 ) | 36.20 ( 136.79 ) | 36.16 ( 13.50) | 5.18 ( 0.87 ) |
|  | Vomiting | 3 | 1.08 ( 0.35 - 3.36 ) | 1.08 ( 0.02 ) | 1.08 ( 0.35) | 0.11 ( -1.37 ) |
| **Suvorexant** | Drug ineffective | 734 | 12.76 ( 11.79 - 13.80 ) | 10.99 ( 6727.67 ) | 10.94 ( 10.12) | 3.45 ( 3.32 ) |
|  | Nightmare | 167 | 85.91 ( 73.45 - 100.48 ) | 83.00 ( 13116.33 ) | 80.46 ( 68.79) | 6.33 ( 5.54 ) |
|  | Somnolence | 158 | 10.61 ( 9.06 - 12.44 ) | 10.30 ( 1326.03 ) | 10.27 ( 8.76) | 3.36 ( 3.04 ) |
|  | Insomnia | 124 | 7.03 ( 5.88 - 8.41 ) | 6.88 ( 623.59 ) | 6.86 ( 5.74) | 2.78 ( 2.45 ) |
|  | Feeling abnormal | 109 | 6.58 ( 5.44 - 7.96 ) | 6.46 ( 503.25 ) | 6.44 ( 5.33) | 2.69 ( 2.34 ) |
|  | Abnormal dreams | 89 | 58.21 ( 47.09 - 71.95 ) | 57.17 ( 4807.38 ) | 55.96 ( 45.27) | 5.81 ( 4.81 ) |
|  | Headache | 88 | 2.49 ( 2.02 - 3.08 ) | 2.46 ( 76.99 ) | 2.46 ( 1.99) | 1.30 ( 0.97 ) |
|  | Hallucination | 87 | 10.87 ( 8.79 - 13.44 ) | 10.69 ( 762.55 ) | 10.65 ( 8.61) | 3.41 ( 2.95 ) |
|  | Wrong technique in product usage process | 80 | 4.47 ( 3.58 - 5.58 ) | 4.41 ( 211.61 ) | 4.41 ( 3.53) | 2.14 ( 1.76 ) |
|  | Dizziness | 79 | 1.76 ( 1.41 - 2.19 ) | 1.74 ( 25.23 ) | 1.74 ( 1.39) | 0.80 ( 0.46 ) |
|  | Delirium | 64 | 13.10 ( 10.23 - 16.78 ) | 12.94 ( 702.47 ) | 12.88 ( 10.06) | 3.69 ( 3.08 ) |
|  | Poor quality sleep | 61 | 40.43 ( 31.35 - 52.15 ) | 39.94 ( 2281.56 ) | 39.35 ( 30.51) | 5.30 ( 4.23 ) |
|  | Fatigue | 59 | 0.97 ( 0.75 - 1.26 ) | 0.97 ( 0.05 ) | 0.97 ( 0.75) | -0.04 ( -0.41 ) |
|  | Sleep disorder | 53 | 12.51 ( 9.53 - 16.40 ) | 12.38 ( 552.29 ) | 12.33 ( 9.40) | 3.62 ( 2.95 ) |
|  | Anxiety | 52 | 4.28 ( 3.25 - 5.62 ) | 4.24 ( 129.02 ) | 4.24 ( 3.22) | 2.08 ( 1.60 ) |
|  | Nausea | 48 | 0.84 ( 0.63 - 1.11 ) | 0.84 ( 1.52 ) | 0.84 ( 0.63) | -0.26 ( -0.67 ) |
|  | Drug effect incomplete | 48 | 19.91 ( 14.96 - 26.48 ) | 19.72 ( 847.05 ) | 19.58 ( 14.72) | 4.29 ( 3.41 ) |
|  | Middle insomnia | 46 | 31.61 ( 23.60 - 42.33 ) | 31.32 ( 1334.66 ) | 30.96 ( 23.12) | 4.95 ( 3.82 ) |
|  | Fall | 44 | 0.90 ( 0.67 - 1.22 ) | 0.90 ( 0.45 ) | 0.90 ( 0.67) | -0.14 ( -0.57 ) |
|  | Overdose | 39 | 5.26 ( 3.83 - 7.21 ) | 5.22 ( 133.08 ) | 5.21 ( 3.80) | 2.38 ( 1.78 ) |
|  | Product use issue | 39 | 2.92 ( 2.13 - 4.00 ) | 2.90 ( 48.77 ) | 2.90 ( 2.12) | 1.54 ( 1.01 ) |
|  | Palpitations | 38 | 4.80 ( 3.49 - 6.61 ) | 4.77 ( 113.19 ) | 4.76 ( 3.46) | 2.25 ( 1.65 ) |
|  | Adverse event | 37 | 7.68 ( 5.55 - 10.61 ) | 7.62 ( 212.56 ) | 7.61 ( 5.50) | 2.93 ( 2.22 ) |
|  | Dyspnoea | 37 | 0.64 ( 0.46 - 0.88 ) | 0.64 ( 7.66 ) | 0.64 ( 0.46) | -0.65 ( -1.10 ) |
|  | Diarrhoea | 35 | 0.55 ( 0.40 - 0.77 ) | 0.56 ( 12.56 ) | 0.56 ( 0.40) | -0.85 ( -1.31 ) |
|  | Product dose omission issue | 32 | 2.27 ( 1.61 - 3.22 ) | 2.27 ( 22.70 ) | 2.27 ( 1.60) | 1.18 ( 0.62 ) |
|  | Malaise | 30 | 0.80 ( 0.56 - 1.14 ) | 0.80 ( 1.50 ) | 0.80 ( 0.56) | -0.32 ( -0.83 ) |
|  | Initial insomnia | 29 | 41.23 ( 28.54 - 59.57 ) | 40.99 ( 1114.15 ) | 40.37 ( 27.95) | 5.34 ( 3.59 ) |
|  | Tremor | 29 | 2.07 ( 1.44 - 2.98 ) | 2.06 ( 15.88 ) | 2.06 ( 1.43) | 1.04 ( 0.46 ) |
|  | Agitation | 28 | 5.86 ( 4.04 - 8.50 ) | 5.83 ( 111.89 ) | 5.82 ( 4.01) | 2.54 ( 1.78 ) |
| **Daridorexant** | Drug ineffective | 66 | 6.53 ( 5.08 - 8.41 ) | 6.07 ( 283.36 ) | 6.07 ( 4.72) | 2.60 ( 2.13 ) |
|  | Nightmare | 42 | 132.78 ( 97.19 - 181.39 ) | 125.77 ( 5160.51 ) | 124.80 ( 91.36) | 6.96 ( 4.55 ) |
|  | Headache | 33 | 5.90 ( 4.16 - 8.36 ) | 5.70 ( 128.64 ) | 5.69 ( 4.02) | 2.51 ( 1.82 ) |
|  | Fatigue | 32 | 3.35 ( 2.35 - 4.77 ) | 3.25 ( 50.59 ) | 3.25 ( 2.28) | 1.70 ( 1.09 ) |
|  | Insomnia | 30 | 10.61 ( 7.37 - 15.28 ) | 10.25 ( 251.06 ) | 10.24 ( 7.11) | 3.36 ( 2.45 ) |
|  | Somnolence | 24 | 9.89 ( 6.59 - 14.85 ) | 9.62 ( 185.89 ) | 9.62 ( 6.40) | 3.27 ( 2.25 ) |
|  | Hallucination | 20 | 15.48 ( 9.93 - 24.14 ) | 15.12 ( 263.84 ) | 15.10 ( 9.69) | 3.92 ( 2.54 ) |
|  | Feeling abnormal | 17 | 6.31 ( 3.90 - 10.21 ) | 6.20 ( 74.37 ) | 6.20 ( 3.83) | 2.63 ( 1.58 ) |
|  | Nausea | 17 | 1.85 ( 1.14 - 2.99 ) | 1.83 ( 6.48 ) | 1.83 ( 1.13) | 0.87 ( 0.12 ) |
|  | Wrong technique in product usage process | 15 | 5.18 ( 3.11 - 8.63 ) | 5.10 ( 49.58 ) | 5.10 ( 3.06) | 2.35 ( 1.29 ) |
|  | Abnormal dreams | 12 | 47.37 ( 26.76 - 83.84 ) | 46.66 ( 534.84 ) | 46.53 ( 26.29) | 5.54 ( 2.56 ) |
|  | Middle insomnia | 11 | 46.42 ( 25.58 - 84.24 ) | 45.79 ( 480.69 ) | 45.66 ( 25.16) | 5.51 ( 2.43 ) |
|  | Product packaging difficult to open | 10 | 318.53 ( 169.69 - 597.94 ) | 314.51 ( 3065.31 ) | 308.50 ( 164.34) | 8.27 ( 2.53 ) |
|  | Dizziness | 10 | 1.36 ( 0.73 - 2.55 ) | 1.36 ( 0.96 ) | 1.36 ( 0.73) | 0.44 ( -0.48 ) |
|  | Therapeutic product effect incomplete | 10 | 13.95 ( 7.48 - 26.04 ) | 13.79 ( 118.63 ) | 13.78 ( 7.38) | 3.78 ( 1.79 ) |
|  | Product availability issue | 9 | 66.83 ( 34.59 - 129.09 ) | 66.08 ( 574.58 ) | 65.81 ( 34.07) | 6.04 ( 2.22 ) |
|  | Diarrhoea | 9 | 0.88 ( 0.46 - 1.70 ) | 0.88 ( 0.14 ) | 0.88 ( 0.46) | -0.18 ( -1.08 ) |
|  | Loss of personal independence in daily activities | 8 | 14.48 ( 7.21 - 29.07 ) | 14.34 ( 99.29 ) | 14.33 ( 7.14) | 3.84 ( 1.56 ) |
|  | Anxiety | 7 | 3.54 ( 1.68 - 7.45 ) | 3.52 ( 12.65 ) | 3.52 ( 1.67) | 1.81 ( 0.39 ) |
|  | Brain fog | 7 | 64.16 ( 30.44 - 135.22 ) | 63.60 ( 429.64 ) | 63.35 ( 30.06) | 5.99 ( 1.82 ) |
|  | Inappropriate schedule of product administration | 7 | 4.15 ( 1.97 - 8.74 ) | 4.13 ( 16.60 ) | 4.12 ( 1.96) | 2.04 ( 0.54 ) |
|  | Dry mouth | 7 | 5.90 ( 2.80 - 12.42 ) | 5.86 ( 28.23 ) | 5.86 ( 2.78) | 2.55 ( 0.84 ) |
|  | Fall | 6 | 0.76 ( 0.34 - 1.69 ) | 0.76 ( 0.46 ) | 0.76 ( 0.34) | -0.39 ( -1.44 ) |
|  | Palpitations | 6 | 4.67 ( 2.09 - 10.42 ) | 4.64 ( 17.15 ) | 4.64 ( 2.08) | 2.21 ( 0.51 ) |
|  | Depression | 6 | 3.79 ( 1.70 - 8.47 ) | 3.77 ( 12.24 ) | 3.77 ( 1.69) | 1.92 ( 0.34 ) |
|  | Pollakiuria | 6 | 9.08 ( 4.06 - 20.27 ) | 9.02 ( 42.78 ) | 9.01 ( 4.04) | 3.17 ( 0.97 ) |
|  | Sleep paralysis | 6 | 1278.52 ( 555.22 - 2944.07 ) | 1268.81 ( 7044.77 ) | 1176.05 ( 510.72) | 10.20 ( 1.66 ) |
|  | Pruritus | 6 | 1.38 ( 0.62 - 3.09 ) | 1.38 ( 0.63 ) | 1.38 ( 0.62) | 0.46 ( -0.71 ) |
|  | Confusional state | 5 | 1.54 ( 0.64 - 3.72 ) | 1.54 ( 0.95 ) | 1.54 ( 0.64) | 0.62 ( -0.69 ) |
|  | Therapeutic product effect decreased | 5 | 16.96 ( 7.04 - 40.88 ) | 16.86 ( 74.53 ) | 16.84 ( 6.99) | 4.07 ( 1.03 ) |

**Note:** This table lists the top 30 PTs with the highest number of reports (descending by n) associated with five drugs in elderly (≥65 years), along with their signal strengths. Values in parentheses represent the lower limit of the 95% confidence interval (for ROR, IC) or the 5th percentile (EBGM05). Signal criteria were: lower limit of ROR 95% CI > 1, PRR ≥ 2 and χ² ≥ 4, EBGM05 > 2, and IC025 > 0.

**Supplementary Table 4.** Top 20 PTs with positive signals across four statistical methods for the five drugs based on the FAERS database

| **Rank** | **Eszopiclone** | **Zopiclone** | **Lemborexant** | **Suvorexant** | **Daridorexant** |
| --- | --- | --- | --- | --- | --- |
| **1** | Drug ineffective (1415) | Drug ineffective (65) | Drug ineffective (28) | Drug ineffective (734) | Drug ineffective (66) |
| **2** | Insomnia (1009) | Insomnia (21) | Nightmare (18) | Nightmare (167) | Nightmare (42) |
| **3** | Dysgeusia (598) | Product quality issue (20) | Somnolence (16) | Somnolence (158) | Headache (33) |
| **4** | Middle insomnia (471) | Completed suicide (16) | Sleep paralysis (8) | Insomnia (124) | Fatigue (32) |
| **5** | Initial insomnia (313) | Product substitution issue (13) | Seizure (8) | Feeling abnormal (109) | Insomnia (30) |
| **6** | Somnolence (172) | Anxiety (7) | Abnormal dreams (7) | Abnormal dreams (89) | Somnolence (24) |
| **7** | Drug effect decreased (76) | Somnolence (7) | Hallucination (7) | Hallucination (87) | Hallucination (20) |
| **8** | Paradoxical drug reaction (59) | Drug effect decreased (7) | Middle insomnia (6) | Wrong technique in product usage process (80) | Feeling abnormal (17) |
| **9** | Hallucination (42) | Dysgeusia (6) | Altered state of consciousness (6) | Delirium (64) | Wrong technique in product usage process (15) |
| **10** | Dry mouth (40) | Sleep disorder (6) | Delirium (6) | Poor quality sleep (61) | Abnormal dreams (12) |
| **11** | Nightmare (36) | Dementia (5) | Pneumonia aspiration (5) | Sleep disorder (53) | Middle insomnia (11) |
| **12** | Nervousness (36) | Tremor (5) | Interstitial lung disease (5) | Anxiety (52) | Product packaging difficult to open (10) |
| **13** | Abnormal dreams (34) | Dry mouth (5) | Sleep terror (4) | Drug effect incomplete (48) | Therapeutic product effect incomplete (10) |
| **14** | Delirium (32) | Abnormal loss of weight (4) | Intentional overdose (4) | Middle insomnia (46) | Product availability issue (9) |
| **15** | Amnesia (28) | Middle insomnia (3) | Oxygen saturation decreased (4) | Overdose (39) | Loss of personal independence in daily activities (8) |
| **16** | Completed suicide (22) | Psychotic disorder (3) | Sleep disorder (4) | Product use issue (39) | Brain fog (7) |
| **17** | Sleep disorder (20) | Orthostatic hypotension (3) | Suicide attempt (4) | Palpitations (38) | Dry mouth (7) |
| **18** | Restlessness (19) | Therapeutic product effect incomplete (3) | Feeling cold (3) | Adverse event (37) | Palpitations (6) |
| **19** | Hangover (19) | Nightmare (3) | Suicidal ideation (3) | Initial insomnia (29) | Pollakiuria (6) |
| **20** | Anorexia (12) | - | Initial insomnia (3) | Agitation (28) | Sleep paralysis (6) |

**Supplementary TABLE 5.** SOCs distribution of adverse events for the five drugs

| **SOCs** | **Eszopiclone** | **Zopiclone** | **Lemborexant** | **Suvorexant** | **Daridorexant** |
| --- | --- | --- | --- | --- | --- |
| Respiratory, thoracic and mediastinal disorders | 76 (1.1%) | 7 (1.4%) | 19 (4.8%) | 108 (2.2%) | 17 (2.2%) |
| Investigations | 102 (1.5%) | 9 (1.8%) | 21 (5.3%) | 76 (1.6%) | 19 (2.4%) |
| Cardiac disorders | 43 (0.6%) | 9 (1.8%) | 8 (2.0%) | 84 (1.7%) | 12 (1.5%) |
| Reproductive system and breast disorders | 18 (0.3%) | 0 (0.0%) | 1 (0.3%) | 7 (0.1%) | 1 (0.1%) |
| Surgical and medical procedures | 12 (0.2%) | 2 (0.4%) | 1 (0.3%) | 13 (0.3%) | 4 (0.5%) |
| Neoplasms benign, malignant and unspecified (incl cysts and polyps) | 14 (0.2%) | 3 (0.6%) | 1 (0.3%) | 20 (0.4%) | 1 (0.1%) |
| Metabolism and nutrition disorders | 38 (0.6%) | 10 (2.0%) | 8 (2.0%) | 40 (0.8%) | 3 (0.4%) |
| Gastrointestinal disorders | 293 (4.4%) | 30 (6.0%) | 22 (5.5%) | 211 (4.3%) | 52 (6.6%) |
| Injury, poisoning and procedural complications | 150 (2.2%) | 45 (9.0%) | 25 (6.3%) | 557 (11.4%) | 55 (7.0%) |
| Infections and infestations | 42 (0.6%) | 5 (1.0%) | 10 (2.5%) | 60 (1.2%) | 10 (1.3%) |
| Vascular disorders | 35 (0.5%) | 11 (2.2%) | 2 (0.5%) | 25 (0.5%) | 4 (0.5%) |
| Nervous system disorders | 1223 (18.3%) | 53 (10.6%) | 81 (20.4%) | 722 (14.8%) | 131 (16.6%) |
| Ear and labyrinth disorders | 23 (0.3%) | 1 (0.2%) | 2 (0.5%) | 19 (0.4%) | 6 (0.8%) |
| Psychiatric disorders | 2365 (35.3%) | 102 (20.3%) | 93 (23.4%) | 1137 (23.3%) | 195 (24.7%) |
| Eye disorders | 61 (0.9%) | 9 (1.8%) | 0 (0.0%) | 39 (0.8%) | 4 (0.5%) |
| General disorders and administration site conditions | 1894 (28.3%) | 117 (23.3%) | 74 (18.6%) | 1381 (28.3%) | 189 (23.9%) |
| Skin and subcutaneous tissue disorders | 99 (1.5%) | 16 (3.2%) | 5 (1.3%) | 84 (1.7%) | 15 (1.9%) |
| Blood and lymphatic system disorders | 6 (0.1%) | 5 (1.0%) | 0 (0.0%) | 8 (0.2%) | 2 (0.3%) |
| Endocrine disorders | 5 (0.1%) | 0 (0.0%) | 0 (0.0%) | 5 (0.1%) | 1 (0.1%) |
| Immune system disorders | 4 (0.1%) | 1 (0.2%) | 0 (0.0%) | 14 (0.3%) | 6 (0.8%) |
| Social circumstances | 12 (0.2%) | 2 (0.4%) | 0 (0.0%) | 16 (0.3%) | 11 (1.4%) |
| Hepatobiliary disorders | 6 (0.1%) | 4 (0.8%) | 3 (0.8%) | 18 (0.4%) | 2 (0.3%) |
| Renal and urinary disorders | 48 (0.7%) | 11 (2.2%) | 8 (2.0%) | 61 (1.3%) | 12 (1.5%) |
| Musculoskeletal and connective tissue disorders | 74 (1.1%) | 8 (1.6%) | 12 (3.0%) | 105 (2.2%) | 13 (1.6%) |
| Product issues | 48 (0.7%) | 42 (8.4%) | 1 (0.3%) | 62 (1.3%) | 25 (3.2%) |

**Note:** This table presents the distribution counts and proportions of adverse events reported for the five study drugs in elderly patients (≥65 years), categorized by MedDRA System Organ Class. Values in parentheses represent the percentage (%) of reports in that SOC category relative to the total number of reports for that drug. Note that a single adverse event report may involve more than one SOC category; therefore, the sum of reports across SOCs exceeds the total number of unique case reports for each drug. SOC: System Organ Class.
